# Concomitant caries and calculus formation from *in situ* dentin caries model

**DOI:** 10.12688/f1000research.2-3.v1

**Published:** 2013-01-04

**Authors:** Frederico B de Sousa, Pablo J Mangueira, David R Tames, Sandra S Vianna, Neriede S Santos-Magalhaes

**Affiliations:** 1 Departmento de Morfologia, Centro de Ciências da Saúde, Universidade Federal da Paraíba, Cidade Universitária, 58051-900, João Pessoa, Paraíba, Brazil; 2 Faculdade de Odontologia, Universidade do Vale do Itajaí, Itajaí, 88302-202, Santa Catarina, Brazil; 3 Departamento de Física, Centro de Ciências Exatas e da Natureza, Universidade Federal de Pernambuco, Cidade Universitária, 50670-901, Recife, Pernambuco, Brazil; 4 Laboratório de Imunopatologia Keizo-Asami (LIKA), Centro de Ciências Biológicas, Cidade Universitária, 50670-901, Recife, Pernambuco, Brazil

## Abstract

The aim of this study was to test the possibility of the concomitant formation of calculus deposits and caries from *in situ* dentin caries model for short time periods. Six volunteers wore palatal removal appliances with four polished dentin specimens protected from intra-oral mechanical forces for up to 14 days. Each volunteer applied a 50% sucrose solution (four times a day) on the specimens and performed a daily mouthwash with 0.05% NaF. Samples were removed after 2, 5, 9 and 14 days *in situ*. Demineralization was analyzed by stereomicroscopy and SEM (secondary electrons and backscattered electrons modes) and calculus was analyzed by energy dispersive spectroscopy and fluorescence spectroscopy. Seventeen samples, at least one sample from each volunteer, presented dental calculus on both carious and non-carious ones, detected in all time intervals. Ca/P ratios of dental calculus ranged from 1.1 to 1.7. Some large calculus deposits on carious surfaces were confirmed by fluorescence. In conclusion, concomitant caries and calculus formation can be found in dentin caries formed *in situ*. This has important repercussions on the study of surface phenomena on the interface between hard dental tissues and dental plaque.

## Introduction

*In situ* caries models are not expected to give rise to calculus formation. However, the formation of loosely mineralized deposits in dental plaque concomitantly with carious dissolution of enamel, in an environment with fluoridated mineralizing solution, has been reported ^1^. In a morphological study with transmission electron microscopy, those deposits were shown not to be identical to calculus as they were never present within intact bacterial cells ^2^. In addition, it was shown by scanning electron microscopy (SEM) that calculus (firmly bound to hard dental tissue) could develop on dentin specimens subjected to an *in situ* carious attack with a daily application of a 50% sucrose solution in individuals living in a water-fluoridated area for periods of four weeks ^3^. While demineralization is likely in such models, no clear-cut evidence was presented. In this study, some samples with calculus had the cariogenic challenge disturbed by weekly mechanical plaque removal, raising the hypothesis that the concomitant development of caries and calculus may occur during shorter periods under the given conditions.

The aim of this study is to report concomitant formation of calculus deposits and caries on dentin surfaces submitted to an *in situ* caries model (using sucrose and fluoride) for short time periods (2–14 days).

## Material and Methods

### Experimental design

Human dentin specimens (3 × 3 mm) from non-erupted third molars, previously autoclavated, had their surfaces consecutively polished with slurries of decreasing grain size until the alumina was 1 mm. Then, they were mounted in customized hand-made resinous removable palatal appliances (thin resinous removable plates placed on the hard palate only and extending from the area close to the central incisors to area close to the first molars), each one containing four specimens distributed in two pairs each in a different side of the appliance, where they were protected from intra-oral mechanical forces by means of plastic perforated tape. A space of up to 2 mm was left between the specimen and the tape. In the mouth, specimens were located near the palatal surfaces of the first permanent molar and the first premolar.

Six volunteers, aged 20–29 years, were selected on the basis of the following criteria: (i) to be otherwise healthy (no medication regimen), (ii) no calculus treatment in the last two years and no clinical calculus deposits at the time of the study, and (iii) to present normal stimulated salivary flow. All volunteers were dental students who lived in non water-fluoridated areas, and were given fluoridated dentifrices (1500 ppm of the same brand) daily. All volunteers provided signed informed consent. Ethical approval was obtained from the Universidade do Vale do Itajaí Human Research Ethics Committee.

Intra-oral periods lasted fourteen days and the volunteers were instructed to use the appliances all day long, except during meals. Two drops of a 50% sucrose solution (the same applied previously) ^3^ was applied onto the specimens only, four times a day to mimic meals, and a 0.05% NaF solution was provided to perform one daily mouthwash for about one minute with the appliance in the mouth (at a different time to the sucrose rinsing). Instructions were given to replace the appliance in the mouth just after the sucrose rinsing and to not brush on the specimens. For each volunteer, specimens were removed after 2, 5, 9 and 14 days of intra-oral period. Six other polished specimens prepared in the same way described above, not exposed to the oral cavity, were also selected for reproducibility during morphological analysis, to make a total of 30 specimens. After the *in situ* period, specimens were stored in 0.1% timol prior to further analysis.

### Scanning electron microscopy (SEM) and energy-dispersive spectroscopy (EDS)

All samples were treated with NaOCl 5% for 15 minutes, to remove organic coatings, then critical point dried with CO _2_-atmosphere of 10 ^-2^ Torr- and coated with a 10 nm-thick layer of gold. The secondary electron detector of a scanning electron microscope JEOL 5900HV (voltage of 15 KeV) was used. Each specimen was analyzed under tilting angles of 0° and 40°. EDS analyses were performed in representative areas by a VOYAGER system attached to the microscope using the following parameters: spatial resolution of 1 mm, electron beam area of 10 mm, working distance of 8–9 mm, voltage of 15 KeV and a live time of 60 s. Ca/P (mol/mol) ratios (mean of 5 measurements) were analyzed using a standardless method - intensity of the standard calculated on the basis of the spectral properties of the sample ^4^-with corrections for atomic number, absorption and fluorescence (ZAF correction). SEM analysis of surface changes (demineralization and dental calculus) was performed twice by blinded examiners with a time interval of seven days between the two analyses. A set of 3 images from each sample was chosen. The Kappa test was used to calculate agreement. Only Schroeder’s type A dental calculus mineralization centers (with mineralized bacterial bodies outlines) ^5^ were considered.

### Stereomicroscopy

After SEM, all samples were treated with NaOCl 5% for 15 minutes to remove the gold coating and then hemi-sectioned centrally on the exposed surface. The surfaces created after cutting were gently polished with alumina 10 mm and then analyzed under the stereomicroscope (Leica MZ12, Leica, Switzerland). Image analysis for the automated identification of demineralized areas and lesion depth measurements were performed using Leica QWIN Plus software (Leica, Switzerland). A standard margin of color discrepancy detection in the software was used for all samples when the software was asked to differentiate sound and carious dentin. The only influence of the operator was to choose a 1 mm-wide central area for automated lesion depth measurement.

### Fluorescence spectroscopy and backscattered scanning electron microscopy (BSE-SEM)

In samples that presented thick (> 100 mm in height) and extensive calcified deposits, a scalpel was used to remove the surface by scraping after stereomicroscopy. The scraped material was dissolved in a solution of HCl 27% (~ 0.2 ml), dispensed in a quartz cuvette and submitted to fluorescence spectroscopy in a commercial photon-counting spectrofluorometer (PC1, ISI, USA and Vinci software, ISI, USA) equipped with a xenon arc lamp operating at 10 mA, using 1 mm slits (bandwidth of 8 nm). Excitation wavelength (λ _excit_) was 416 nm and the emission (λ _emis_) was collected from 425 to 800 nm (mean of 15 readings). Fluorescence of the other two saturated solutions in HCl 27%, one with human dental calculus and another with human dentin, were also analyzed.

Next, all samples had their cut surface prepared (polished, dried and metallic coated as described above) for SEM analysis using a backscattered electron detector (voltage of 15 KeV) in order to confirm the *presence* of demineralization. During this latter procedure, the demineralized layer of most samples broke apart so that only the inner parts remained for BSE-SEM analysis.

### Statistical analysis

The differences between *in situ* periods of all volunteers with regard to lesion depth were evaluated by paired t test (significance limit at 5%).

## Results and discussion

The occurrence of demineralization and calcified deposits was depicted from SEM examination (Table 1). Kappa’s coefficients of intra-examiner agreement were 0.88 and 0.83, and 0.78 for inter-examiner agreement of the diagnosis of demineralization with SEM. For dental calculus, it was decided to count only cases with full agreement between examiners. Mean lesion depth values were 0.0, 0.197 (± 0.059), 0.442 (± 0.062) and 1.100 mm (± 0.212) for 2, 5, 9 and 14 *in situ* periods, respectively (differences were statistically significant, p < 0.05). BSE-SEM analysis confirmed the presence of demineralization (lower gray levels due to lower mean atomic number) detected by stereomicroscopy.

**Table 1.** Occurrence of demineralization and calculus deposits (with mean of 5 Ca/P ratios obtained by EDS-SEM). * Samples with 50% or more of their surfaces covered by calculus.

**Volunteer**	**Demineralization**	**Dental calculus (Ca/P, mol/mol)**
I	5, 9 and 14 days	5 days (1.04 ± 0.03)
II	2, 5, 9 and 14 days	2 (1.12 ± 0.04), 9 (1.40 ± 0.02) and 14* days (1.53 ± 0.04)
III	5, 9 and 14 days	2 (1.49 ± 0.07), 5 (1.48 ± 0.05) and 9* days (1.43 ± 0.06)
IV	5, 9 and 14 days	2 (1.47 ± 0.02), 5* (1.52 ± 0.04), 9* (1.66 ± 0.06) and 14 days (1.73 ± 0.05)
V	5, 9 and 14 days	2 (1.38 ± 0.02), 5 (1.51 ± 0.05), 9* (1.54 ± 0.05) and 14* (1.71 ± 0.06) days
VI	2, 5, 9 and 14 days	5 (1.56 ± 0.05) and 14* (1.41 ± 0.04) days

Seventeen samples presented calculus deposits (14 with demineralization; Table 1). Seven of these presented ~ 50% or more of the surface area covered by the deposits. In the other samples, deposits formed isolated or connected islands of 50–500 mm wide. Ca/P ratios resemble the structures of dicalcium phosphate (Ca/P of 1.0), octacalcium phosphate (Ca/P of 1.3), magnesium whitlockite (Ca/P of 1.5) and hydroxyapatite (Ca/P of 1.67–2.3) ^5– 7^ (Table 1). Mean Ca/P ratio of dentin (for all samples) was 1.88 mol/mol (± 0.22), compatible with hydroxyapatite. Demineralization surrounding calculus deposits occurred in thirteen samples.

The crystalline structure of young calculus is reported to be mainly of dicalcium phosphate in the early stages, after which octacalcium phosphate develops and, with further growth, whitlockite and hydroxyapatite are formed ^5^. The driving force for the mineralization of dental plaque is the supersaturation of plaque fluid with respect to calcium phosphate, which is pH- and temperature-dependent. It is known that dicalcium phosphate and octalcacium phosphate can be precipitated in acidic environments whereas hydroxyapatite is dissolved ^7, 8^. Magnesium whitlockite or simply whitlockite (a type of calcium phosphate where calcium is partly substituted by magnesium) has a structure that is not easily distinguished from β-tricalcium phosphate or magnesium-containing β-tricalcium phosphate, and this is the reason why they have been referred as synonyms ^9^. However, it is known that they are distinguishable by careful powder diffractometry ^10^, although both present a Ca/P ratio of 1.5 ^7^. While β-tricalcium phosphate does not form in biological systems, whitlockite is found in many biological mineralizations ^9^. Whitlockite precipitates at low, neutral or high pH, with magnesium stimulating whitlockite precipitation at the expense of hydroxyapatite ^7^. The presence of hydroxyapatite most probably reflects pH fluctuations (with either neutral or high pH events) during the periods of time employed. In this context, it is possible to chemically explain the concomitant calculus and caries formation in our model.

Crystallite morphology and Ca/P ratios resembling hydroxyapatite in 10-day old calculus deposits have been reported previously ^11^.

**Figure 1.**
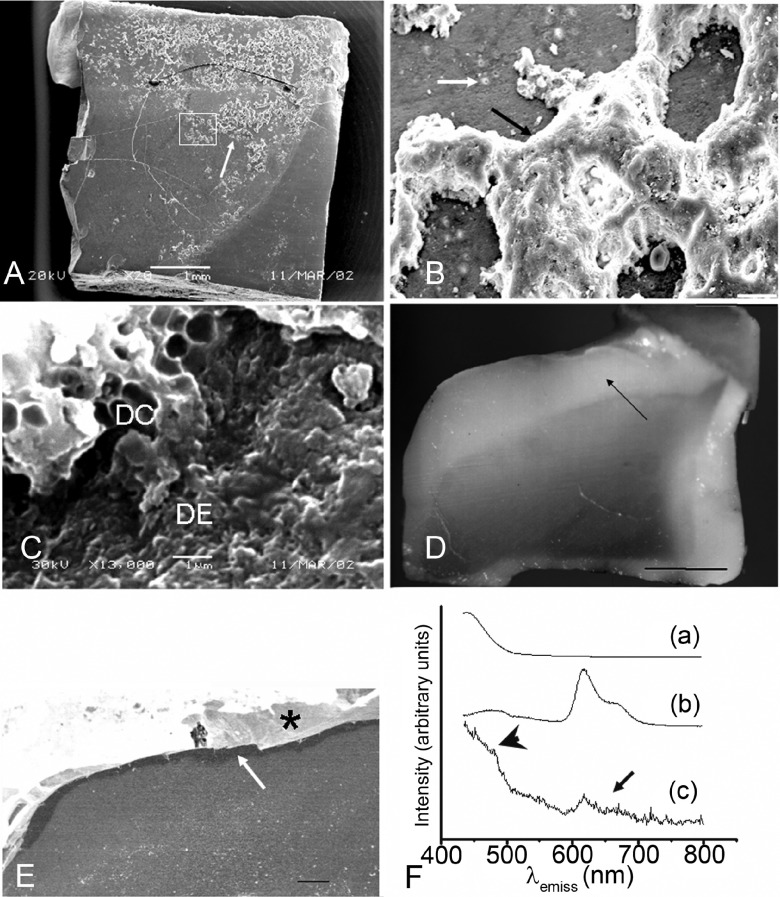
**A**, calculus deposits (arrow), of macroscopic size, on a 9-day sample with demineralization (Bar = 1 mm). **B**, detail of the area outlined in “A”, showing opening of dentinal tubules (white arrow) and dental calculus (black arrow) (Bar = 20 mm). **C**, detail of the area close to the white arrow in “B”, showing mineralized bacterial outlines delimitating spaces of ~ 1 mm in diameter (DC, dental calculus) and the demineralized dentin (DE) (Bar = 1 mm). **D**, histological aspect of the same sample after hemi-sectioning showing demineralization (black arrow) below the experimental surface (Bar = 1 mm). Opaque outline is demineralization caused by bacterial acid infiltrated around the sample. **E**, BSE-SEM image (bar = 300 mm) showing, below the fractured surface (asterisk), a dark layer (white arrow) representing the preserved part of the demineralised area indicated by the black arrow in “D”. **F**, fluorescence spectroscopy data of different samples dissolved in HCl 27% and excited with light at 416 nm: (a), human dentin; (b), human dental calculus; and (c), sample from the surface of the sample shown in “A”, showing fluorescence of dentin (arrowhead) and dental calculus (arrow).

As the *in situ* period proceeded, calculus deposits (resembling the morphology of Schroeder's type A calculus deposits ^5^) with increased height, macroscopic in some cases, were seen on demineralized areas (Figure 1A-C). The shapes of intact bacterial bodies (cocci, filaments and rods) remained within extracellular calcified trabeculae, indicating that the mineralization process was preferably extracellular. Some deposits were identified only after tilting the sample to 40°. No bacterial cell remnants were identified within calculus deposits.

Three samples (from two volunteers; *in situ* times of 9 and 14 days) that presented “large” deposits of calculus and caries were submitted to fluorescence spectroscopy. All of them presented fluorescence of dental calculus ( Figure 1F: the same sample of Figs. 1A-E) and histological demineralization (Figure 1C-E). The dark layer shown in Figure 1E represents demineralization (preserved part after polishing) seen under BSE-SEM.

Fluorescence of human dental calculus in acid solution has been shown to present the highest emission with λ _excit_ at 416 nm and λ _emis_ peaks at 620 and 660 nm (originated from hematoporphyrin) ^12^. A lower fluorescence emission band below 580 nm has also been reported for human dental calculus ^13^. Human dentin fluoresces with a λ _emis_ peak at 440 nm and does not present any emissions in the region of 580–700 nm ^14^, which is in accordance with our fluorescence data for λ _excit_ at 416 nm (Figure 1F). The selected samples with “large” calcified deposits (deposits < 100 µm in height cannot be detected by a spectrofluorometer system with a lamp source as excitation light ^15^) showed a mixture of fluorescence bands of human dental calculus and human dentin (Figure 1F). The double treatment with NaOCl 5% and the absence of bacterial cell remnants during SEM examination exclude the influence of loosely bound organic material of bacteria on the observed fluorescence.

The concomitant development (based on our time intervals) of caries lesions and calculus deposits using an *in situ* caries model reported here occurred under conditions known to cause the formation and growth of dental plaque. Combining SEM, fluorescence spectroscopy and stereomicroscopy (Figs 1A-F), we have shown caries and calculus developments in the same dentin sample that was originally non-carious and had not been exposed to the oral cavity before the *in situ* experiment. Our model was able to promote time-dependent demineralisation (seen from lesion depth data), which means that volunteers followed instructions properly. To our knowledge, this is the first time that this phenomenon has been reported on the basis of the combination of such mutually validating techniques.

## Conclusion

In conclusion, calculus formation in active cariogenic dental plaque has important repercussions on the study of surface phenomena on the interface between hard dental tissues and dental plaque. It is also important in the development and the evaluation of anti-tartar and caries-preventative agents. Our study shows that more attention should be paid by those studying *in situ* dentin caries to the identification of calculus on hard dental tissues exposed to *in situ* caries models.
