# Supplementary material for: Concomitant caries and calculus formation from in situ dentin caries model
Source: F1000Res. 2013 Jan 4;2:3. [Version 1] doi: 10.12688/f1000research.2-3.v1 (PMC3752729; doi:10.12688/f1000research.2-3.v1)
Supplement: Peer review history

# Peer review: Referee response for version 1

**Published:** 2013-04-23

**Authors:** Neville Gully

**Affiliations:** School of Dentistry, The University of Adelaide, Adelaide, Australia

It was noted that all dentin samples were exposed to sucrose solutions, fluoride and were protected by tape. Only the unexposed slabs were used as controls and none of these three conditions were controlled. A more complete picture would have been possible with their inclusion.

The authors claimed “It is known that dicalcium phosphate and octacalcium phosphate can be precipitated in acidic environments whereas hydroxyapatite is dissolved.” They listed references 7 and 8 to support this statement. However, I was unable to access reference 7 and could not find supporting data in reference 8. While DCP is slightly less soluble than HAP around pH4 I would not consider that to mean that either DCP or OCP readily precipitate at that pH.

I have read this submission. I believe that I have an appropriate level of expertise to confirm that it is of an acceptable scientific standard.

# Response

**Published:** 2013-05-20

**Authors:** Frederico Sousa

**Affiliations:** Federal University of Paraiba, Brazil

I thank the reviewer for his valuable comments and his opinion to accept the article is greatly acknowledged by us. 

Regarding the first paragraph of the reviewer’s comment, we understood that the critique was that some control was lacking. We think that the inclusion of samples that were exposed to the oral environment but did not develop caries would no add relevant information for the aim of the study. The in situ caries induction aimed at producing caries lesions with known history and with severity directly proportional to the in situ time. Mean lesion depth increased with in situ time, confirming the prediction. In an environment where caries develops, surface features of the samples were analyzed in order to verify whether calculus deposits could have been formed under the same conditions. Such conditions are those common to in situ caries models. Because the length of the article is short, we focused on the more relevant data (samples with dental calculus). If we had exposed dentin samples that remained sound after the in situ period, the presence of absence of calculus deposits on those samples would have not contributed to prove that calculus deposits can be formed concomitantly with caries lesions in short time intervals in an in situ caries model. 

In the case of precipitation of dicalcium phosphate (DCP) and octacalcium phosphate (OCP) in acidic environments, Johnsson *et al.* 1992 describes evidence indicating that formation of both DCP and OCP *in vivo*, during normal mineralization, is quite different from the conditions operating under pathological mineralization. The authors cite reports of the precipitation of DCP and OCP in slightly acidic conditions (please see last paragraph of the second column on page 78 of Johnsson *et al.* 1992). The critical pH of dentin is between 6.5 and 7 ( Hoppenbrouwers *et al.* 1987). There is a pH range below the critical pH of dentin that can be considered as slightly acidic, and so it is consistent with the possible precipitation of both DCP and OCP in our samples. Regarding formation of DCP and OCP in acidic conditions *in vitro*, evidence can be found in the following papers; De Rooij *et al.* 1984 and Lundager Madsen *et al.* 1991.

# Peer review: Referee response for version 1

**Published:** 2013-01-21

**Authors:** Leonard Crocombe

**Affiliations:** Australian Research Centre for Population Oral Health and the School of Dentistry, The University of Adelaide, Adelaide, Australia

I have read this submission. I believe that I have an appropriate level of expertise to confirm that it is of an acceptable scientific standard.
